# A caloritronics-based Mott neuristor

**DOI:** 10.1038/s41598-020-61176-y

**Published:** 2020-03-09

**Authors:** Javier del Valle, Pavel Salev, Yoav Kalcheim, Ivan K. Schuller

**Affiliations:** 1 Department of Physics and Center for Advanced Nanoscience, University of California-San Diego, La Jolla, California, 92093 USA; 2 Present Address: Department of Quantum Matter Physics, University of Geneva, 24 Quai Ernest-Ansermet, 1211 Geneva, Switzerland

**Keywords:** Electronic properties and materials, Phase transitions and critical phenomena, Electronic properties and materials, Electronic properties and materials, Electronic and spintronic devices

## Abstract

Machine learning imitates the basic features of biological neural networks at a software level. A strong effort is currently being made to mimic neurons and synapses with hardware components, an approach known as neuromorphic computing. While recent advances in resistive switching have provided a path to emulate synapses at the 10 nm scale, a scalable neuron analogue is yet to be found. Here, we show how heat transfer can be utilized to mimic neuron functionalities in Mott nanodevices. We use the Joule heating created by current spikes to trigger the insulator-to-metal transition in a biased VO_2_ nanogap. We show that thermal dynamics allow the implementation of the basic neuron functionalities: activity, leaky integrate-and-fire, volatility and rate coding. This approach could enable neuromorphic hardware to take full advantage of the rapid advances in memristive synapses, allowing for much denser and complex neural networks.

## Introduction

Machine learning has experienced an unprecedented growth in recent years, often referred to as an “artificial intelligence revolution”^1,2^. Its fundamental approach is inspired by biological systems: using neural networks to classify large amounts of data into sorting categories. Classic examples are speech and image recognition^1,2^. Neural networks are composed of two basic elements: neurons and synapses. Current machine learning schemes implement these elements at a software level: neurons and synapses are simulated on standard computers based on a von Neumann architecture^1,2^. This approach is inefficient in terms of computation speed and energy consumption, motivating a search for hardware-based systems that imitate the brain. This idea was initially proposed more than fifty year ago^3–5^, and attained widespread popularity with the works of Carver Mead^6^. Since then, CMOS-based circuitry has been successfully used to realize neuromorphic systems, allowing to build tuneable and efficient neural networks^7–9^. Unfortunately, CMOS-based components rely on combinations of multiple transistors and capacitors that make them complex and large^9^. This limits circuit scalability and, hence, poses a limitation to achieve dense neural networks which could eventually rival the brain.

A solution to this problem might be found in “neuromorphic materials”, whose intrinsic properties mimic those of neurons and synapses^10,11^. Resistive switching (RS), a phenomenon in which an applied electric field modifies the resistance of a material^12–14^, offers a unique opportunity to achieve this goal. RS can be volatile^15–21^ or non-volatile^22,23^, which can be used to emulate neuron or synapse behaviours, respectively. Multiple groups have used RS to achieve synaptic functionalities^24,25^, and memristor crossbar arrays have already been used to perform pattern recognition^26–28^. These synapse realizations, however, still rely on traditional electronics to play the role of neurons (neuristors). This approach does not take full advantage of the scalability and simplicity offered by memristive synapses, and motivates the search for a simpler and more scalable neuristor.

**Figure 1:**
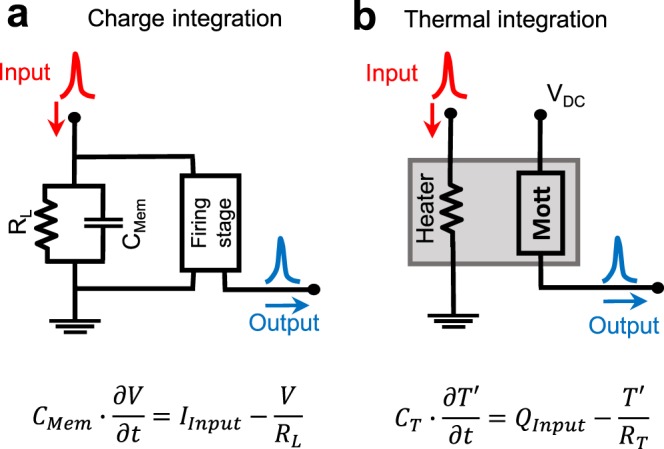
Charge vs thermal signal integration. (**a)** Circuit representation of the leaky integrate-and-fire neuron model. Its dynamics are described by the equation shown below the circuit. A capacitor *C*_*Mem*_ represents the neuron membrane capacitance, and accumulates charge from input current pulses *I*_*Input*_ (red). The leaking resistor *R*_*L*_ represents the leaky term of the equation. If the voltage *V* across *C*_*Mem*_ rises above a threshold, the firing stage will produce an output spike (blue). (**b**) Schematic representation of a thermal transfer-based neuristor. Incoming current pulses (red) dissipate power (*Q*_*Input*_) in a heating resistance. A Mott nanodevice is kept under a DC voltage *V*_*DC*_, and will create a current spike (blue) if a threshold temperature is exceeded. The equation below describes the thermal dynamics of the system, where *C*_*T*_ is the thermal capacitance, *R*_*T*_ the thermal resistance and *T*′ = *T* − *T*_*Eq*_: being *T* the local temperature and *T*_*Eq*_ the equilibrium (substrate) temperature.

A neuristor must feature the most basic functionalities of real neurons^29^: i) leaky integrate-and-fire, ii) activity (outputting a current), iii) volatility (resetting after a firing), and iv) rate coding of the external stimuli. Performing leaky integrate-and-fire is one of the key functionalities of a neuristor. It must sum all the input stimuli coming from previous neurons and fire an output spike when the excitation is above a certain threshold^29^. In the case of biological neurons, the cell membrane acts as a capacitor that integrates incoming ionic currents. The firing mechanism, based on voltage gated sodium and potassium channels, is activated once the membrane potential exceeds a certain threshold. CMOS neuristors use a similar approach (Fig. 1a): a capacitor plays the role of cell membrane by integrating current from incoming pulses^9^, while the CMOS circuitry produces the firing events. Pickett *et al*.^30^, Ignatov *et al*.^31^ and Yi *et al*.^32^ also use capacitive integration, but in their case volatile RS in a Mott insulator is utilized in the firing stage. While this parallelism with biological systems is appealing^33^, the use of capacitors to store the internal state of the neuron limits the circuit scalability. In order to avoid malfunctioning, their capacitance must be much larger than the parasitic capacitance of the electrode lines. Integration capacitors cover a large area in current CMOS neurons, leading to typical sizes in the order of 10–100 μm^9^. Capacitor downscaling is one of the most challenging issues in other technologies such as DRAM, where the industry has dedicated intense effort towards developing complex 3D capacitive structures to circumvent this problem^34^.

Wang *et al*.^35^, Tuma *et al*.^36^ and Stoliar *et al*.^37^ successfully implement integrate and fire dynamics without the use of capacitors by utilizing diffusive memristors, phase-change materials and Mott insulators, respectively. However, these systems are not active as they do not generate a current. Moreover, they are not volatile i.e. do not reset automatically, a characteristic needed for spiking dynamics. This limits their autonomy and their practical implementation as standalone neurons. Yajima *et al*.^38^ introduced a dedicated circuit to reset a Mott integration device. Although their neuristor is capable of performing all basic neuron tasks, the use of multiple operational amplifiers makes this implementation rather complex. A fully autonomous and scalable neuristor is yet to be found.

Instead of electrical currents, we propose using heat flow to perform computing tasks, an approach known as caloritronics. Temperature substitutes charge as the integrating variable, as depicted in Fig. 1b. Current spikes coming from previous neurons induce Joule heating while passing through a resistive element (heater), increasing local temperature with every spike. The heater is thermally coupled to a firing element that is very sensitive to temperature changes and fires once a threshold temperature is exceeded. In this work, we use VO_2_, a well-known correlated oxide with a sharp insulator-to-metal transition (IMT) around 340 K^39^ (Supplementary Figure S1a), as the firing element. We realize leaky integrate-and-fire using the thermal dynamics, which are governed by similar equations to those describing the charge dynamics of a leaky capacitor (see Fig. 1a,b). Adopting local temperature as the internal state allows building simple-design neuristors that can be downsized to the nanoscale, as thermal dynamics equations preserve the same form independent of the system size.

**Figure 2:**
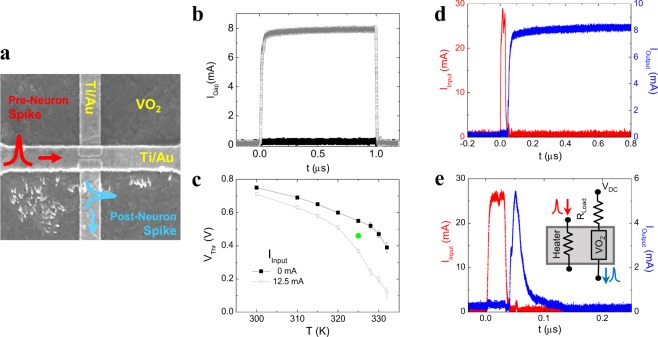
Experimental realization of the proposed neuristor. (**a**) SEM image of the device. Two layers of electrodes, electrically isolated by an Al_2_O_3_ spacer, are visible. Bottom layer consist of two Ti/Au electrodes, running vertically in the image. A small gap (~50 nm) is left in between. The upper electrode is kept at *V*_*DC*_ bias. The top electrode layer is a Ti/Au nanowire, running horizontally in the image. Input current pulses (red) are sent through that heating electrode, while the neuristor output (blue) is collected though the lower bottom electrode. (**b**) Current through a VO_2_ gap vs time, when a 1 μs voltage pulse is applied. *T* = 315 K. Two different pulse amplitudes are shown, 0.87 V (black) and 0.92 V (grey). (**c**) Threshold voltage vs temperature in a gap, when two different DC currents are applied to the heater: 0 mA (black) and 12.5 mA (grey). The green dot is an example of the *V*_*DC*_ and temperature conditions for the device to behave as a neuristor. (**d**) Current vs time. Left axis (red curve) shows the input current through the heater. Right axis (blue curve) shows the output through the gap. Conditions were *V*_*DC*_ = 0.85 *V*_*Th*_ and *T* = 325 K. **e** Current vs time, with a load resistor in series with the gap. Left axis (red) shows the input current pulse. Right axis (blue) shows the output through the gap. *R*_*Load*_ = 10 kΩ, *V*_*DC*_ = 0.88 *V*_*Th*_ and *T* = 325 K. Inset: schematic circuit showing *R*_*Load*_ connected in series with the gap.

We fabricated and tested a proof-of-concept neuristor that performs all basic neuronal functionalities. It consists of a VO_2_ thin film on top of which two layers of electrodes are patterned (the detailed fabrication process can be found in methods section and Supplementary Figure S2). The first layer consists of two Ti/Au electrodes (running vertically in Fig. 2a) separated by a 50 nm gap. These electrodes are used to apply voltage to the VO_2_, and provide the source of the neuristor’s active output. If the voltage is high enough, a transition into the metallic phase can be electrically triggered^15,16,40^ (Supplementary Figure S1b). Figure 2b shows the current as a function of time when a voltage pulse is applied to the gap. It illustrates the threshold nature of the voltage triggered IMT: the device becomes metallic once a threshold voltage (V_Th_) is exceeded^40^. The second electrode layer is a Ti/Au nanowire (running horizontally in Fig. 2a) which acts as a heater. It is separated from the bottom electrodes by a 70-nm-thick Al_2_O_3_ layer which provides electrical insulation (resistance larger than 20 MΩ), but ensures thermal coupling between the nanowire heater and VO_2_ gap.

Figure 2c shows V_Th_ of the VO_2_ gap as a function of temperature. Two cases are shown: zero (black) and 12.5 mA (red) current flowing through the nanowire heater (horizontal electrode). In the second case, Joule heating locally increases the temperature of the gap reducing its V_Th_. To work as a neuristor, the gap is kept under a DC bias just below its threshold voltage (V_DC_ < V_Th_), as represented in Fig. 2c by a green dot. When a high enough current pulse I_Input_ is passed through the heater, it lowers V_Th_ below V_DC_, and the gap turns metallic, generating an output current through the bottom electrodes (I_Output_). This situation is presented in Fig. 2d, where a 30 ns current pulse is applied to the heater, triggering the IMT in the gap. We must emphasize that the output and input electrodes are electrically isolated, and an output current is generated when the neuristor fires, making it an active element (as it must be powered to operate, and outputs power during operation). Our device releases energy when stimulated, a crucial property to avoid a re-amplification stage after each neural layer. This goes one step beyond previous RS-based neuristors^30–32^, which become conducting after performing integrate-and-fire but do not create an output on their own. Furthermore, electrically decoupled input and output of our neuristor would allow building a multilayer neural network without using buffer circuits to prevent current backflow from post-synaptic to pre-synaptic neuristors.

Volatility is a necessary feature to implement spiking dynamics. As presented so far, our device would remain conductive once triggered. To reset the VO_2_ gap after the firing event, we added a resistor (*R*_*Load*_) in series with the gap (inset Fig. 2e). The role of this resistor is to lower the voltage across the gap once the VO_2_ becomes metallic^41–43^, which in turn reduces heat dissipation and decreases local temperature after the firing event. As a result, the VO_2_ returns to its insulating state after firing (Fig. 2e) giving the desired effect: spike in – spike out.

**Figure 3:**
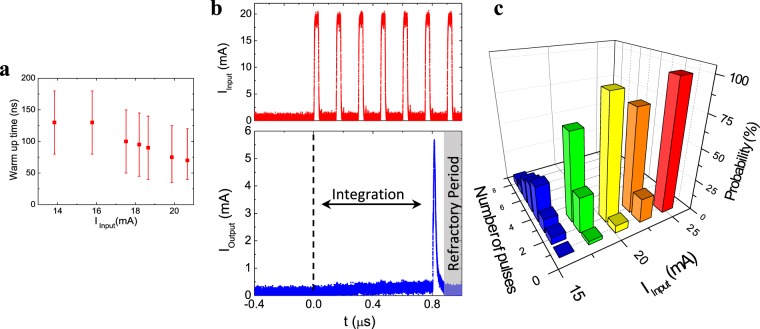
Leaky integrate and fire dynamics. (**a**) Warm up time as a function of the input current. *V*_*DC*_ = 0.85 *V*_*Th*_ and *T* = 325 K. (**b**) Current vs time when the neuristor is stimulated with a train of pulses. Upper panel shows the input current, consisting on 30 ns pulses with a 150 ns period. Bottom panel shows the output current through the gap. There is an integration time between the moment the pulses are applied and the moment the IMT is triggered. *R*_*Load*_ = 10 kΩ, *V*_*DC*_ = 0.88 *V*_*Th*_ and *T* = 325 K. No firing occurs after the last pulse because the neuron is in its refractory period (indicated with a grey shaded area). (**c**) Probability that the device will fire after a certain number of pulses. Pulses are 150 ns apart from each other. Several pulse amplitudes are shown. *V*_*DC*_ = 0.91 *V*_*Th*_ and *T* = 325 K.

Leaky integrate-and-fire (LIF) dynamics are governed by the characteristic thermal times of our device, given by its specific heat and thermal resistance to the substrate. Figure 3a shows the warm up times of the neuristor as a function of I_Input_, that is, how long it takes the device to warm and fire once the current flows through the heater. Typical times are on the order of 10–100 ns. Using pulse widths and rates around that timescale allow us to implement LIF dynamics. Figure 3b shows the response of the neuristor (I_Output_) when a train of current pulses is sent to the heater (I_Input_). The first pulse does not raise the temperature enough to fire the device, but the cumulative effect of several pulses adds up to trigger the IMT after an integration period. The number of pulses necessary to produce a firing event depends on the pulse amplitude. Figure 3c shows the probability of the neuristor firing after a certain number of pulses are applied to the input. Several current amplitudes are shown, offering a clear visualization of the LIF dynamics. For high current pulses, the device is always triggered with just one pulse. For lower currents, the probability of it firing with just one pulse decreases, and more pulses are necessary to induce an output spike. The overall mean integration time is shifted to longer values as the current amplitude is decreased. When the input current is too low, heat leakage into the environment overcomes the dissipated power, and the device does not fire for any number of pulses.

**Figure 4:**
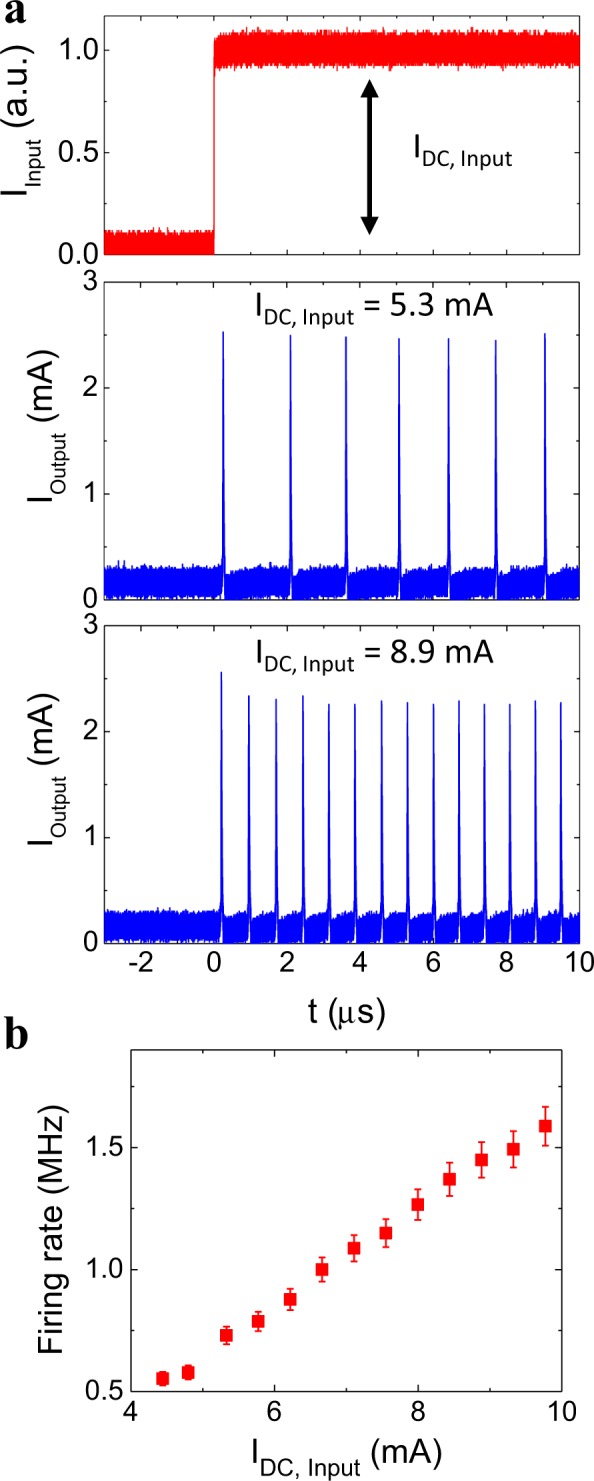
Rate coding. (**a**) Current vs time when a DC current is applied as input. Top panel shows the input current I_DC,Input_ through the heater (red). Middle and lower panels show the output spiking pattern (blue) induced in the gap. The responses to two I_DC,Input_ values are shown: 5.3 mA (middle panel) and 8.9 mA (lower panel). R_Load_ = 10 kΩ, V_DC_ = 0.88 V_Th_ and T = 325 K. (**b**) Firing rate of the output spiking pattern as a function of I_DC,Input_.

Another basic feature of biological neurons is rate coding: the frequency at which a neuron spikes depends on the amplitude of its stimulus. Strong stimuli produce high frequency spiking, while weak stimuli yield slower patterns^29,44^. Our neuristor reproduces that feature, as shown in Fig. 4a. A constant current is passed through the heater, resulting in a repetitive spiking output. The frequency of the output increases with the input current (Fig. 4b). After the neuristor fires, both the temperature and the voltage across the gap drop, leaving the system in a refractory period until they increase back to their initial values.

The duration of the refractory period depends on the interplay between neuristor’s thermal and electrical properties. The electrical charging time is determined by the *RC* constant, being *R* and *C* the resistance and capacitance of the system. While we do not use external capacitive elements, some intrinsic capacitance will always be present due to the experimental set up. The warm up time is given by *R*_*th*_*C*_*th*_, where *R*_*th*_ is the thermal resistance and *C*_*th*_ the thermal capacitance. Whether the refractory dynamics are dominated by thermal or electrical effects depends on the *RC*/*R*_*th*_*C*_*th*_ ratio. Considering the geometry and materials of our device, we estimate *R*_*th*_*C*_*th*_ to be around 10^−8^ s (See methods). Since *R* is in the 10^4^ Ω range, electric parasitic capacitance is expected to be dominant for *C* above 10^−12^ F.

**Figure 5:**
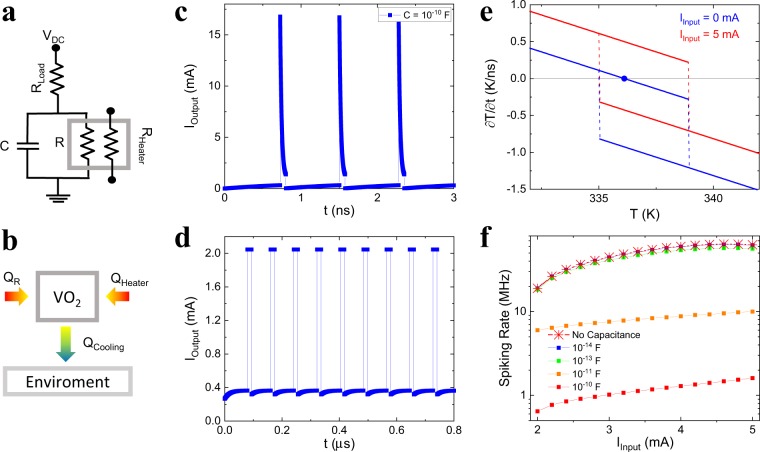
Simulation of the neuristor dynamics. (**a**,**b**) Schematic representation of the simulated electrical (**a**) and thermal (**b**) circuits. V_DC_ is the bias voltage. C is the parasitic capacitance, R is the resistance of the VO_2_ gap, R_Load_ is the resistance of the load resistor and R_Heater_ is the heater resistance. Q_R_ and Q_Heater_ are the powers dissipated in the VO_2_ gap and heater, respectively. Q_Cooling_ is the power lost to the substrate and contact pads though thermal conduction. For a complete description of the model, see the methods section. (**c**,**d**) Output current vs time for C = 10^−10^ F (**c**) and C = 0 (**d**). The neuristor shows spiking behaviour both with and without the series capacitor. (**e**) Thermal stability analysis derived from the model. ∂*T*/∂*t* vs *T* is showed for two different cases, *I*_*Input*_ = 0 mA and *I*_*Input*_ = 5 mA. An external input from the nanoheater is capable to drive the system into a permanent spiking cycle, with no stability points. (**f**) Spiking rate vs I_Input_ for five different capacitance values. Rate coding is observed in all cases.

To gain a better understanding of the interplay between electrical and thermal properties, we performed lumped-element simulations of the neuristor operation. Figure 5a,b show the electrical equivalent circuit and a schematic of the thermal model used in our simulations (see methods section for more details). In the equivalent circuit, we explicitly include the parasitic capacitance associated with the device. The thermal model takes into account the heating provided by both the nanowire heater and the *V*_*dc*_ bias, and the heat loss into the environment. Figure 5c shows *I*_*Output*_ vs *t* when a dc *I*_*Input*_ is applied at *t* = 0, for a system with a relatively large electric capacitance *C* = 10^−10^ F. Repetitive spiking, similar to the experimental result is observed. This suggests that in our particular devices, recovery dynamics during the refractory period are determined mainly by the parasitic capacitance. However, such capacitance is not necessary to produce spiking dynamics. Figure 5d shows *I*_*Output*_ vs *t* when a dc *I*_*Input*_ is applied at *t* = 0, for a system with no parasitic capacitance: spiking behaviour is still observed, although with clear differences in the shape and time separation between the individual spikes. The mechanism behind the spiking dynamics can be understood by considering the stability points of the system^45^. Figure 5e shows time derivative of the temperature, ∂*T*/∂*t*, vs *T* for different *I*_*Input*_. Sharp discontinuities in ∂*T*/∂*t* are present due to the IMT, resulting in a hysteresis curve. When there is no current through the heater, the system stabilizes at a certain temperature and does not oscillate. Adding an input shifts the curve in a way in which the hysteresis jumps discontinuously between ∂*T*/∂*t* > 0 and ∂*T*/∂*t* < 0, so ∂*T*/∂*t* is never equal to zero. This traps the system in a persistent oscillation state, purely due to thermal dynamics. In this way, the spiking behaviour of the VO_2_ gap can be externally controlled with a heat current. Both electrically, *RC* > *R*_*th*_*C*_*th*_, and thermally dominated, *RC* < *R*_*th*_*C*_*th*_, systems produce the rate coding property, as shown in Fig. 5f, where the spiking rate is plotted as a function of *I*_*Input*_ for several values of *C*.

Many of the relevant parameters of the proposed neuristor depend on the particular device design, as well as on the intrinsic properties of the chosen materials. This gives plenty of room to explore and improve its functionalities. Different substrates, insulating spacers or geometric designs will strongly change the device properties. For instance, a smaller device and less thermally conductive substrate and contact pads would reduce heat leakage to the environment, allowing for the use of lower currents. For instance, using Ti instead of Au as metallic contact would drastically reduce heat loss into the pads. Similarly, using TiO_2_ instead of Al_2_O_3_ as a substrate can decrease thermal conductance by a factor of 20. A simple calculation (see methods), shows that by changing the materials, thermal conductance and hence Joule heating can be reduced by more than two orders of magnitude.

Although the currents used in our proof-of-concept device are large, the short duration of each spike (~30 ns) yields an energy consumption of 3.10^−9^ J/spike. By reducing parasitic capacitance and optimizing materials and geometry, this number could be brought down to around 10^−11^ J/spike, which is comparable to the performance of biological neurons. Other design strategies, such as extremely localized Joule heating^46^, could also be used to further decrease energy consumption. Regarding size, our device occupies less than 1 μm^2^, reducing neuron area by more than an order of magnitude compared to biological neurons, and almost four orders compared the most compact silicon neuron circuits^9^.

Another attractive feature of our approach is the potential of signal amplification without needing further elements: since the input and output are electrically isolated, it is possible for the output current to be larger than the input current. This is of fundamental importance; real neural networks propagate signals across several neuron layers, and the signal must be amplified after each layer. Previous neuristor implementations are passive, and therefore the output is always smaller than the input. This makes the use of CMOS based electronics mandatory, partially defeating the purpose of building a purely oxide electronics. We must note that extensive device optimization must be done before this is experimentally possible in a caloritronics-based device. Nevertheless, our lumped-element simulations show that this is a feasible scenario if heat conductance between the heater and the gap is improved. In fact, the results presented earlier in Fig. 5d also demonstrate self-amplification: the device generates a ~2 mA output current out of a 1.3 mA input.

The use of a heat transfer-based device may also come with some drawbacks that must be considered. One of them is crosstalk between neurons which could limit device density. Let’s consider two neuristors placed next to each other. With our current device dimensions, the distance between the two VO_2_ gaps could be as low as 1 μm, comparable to the typical pitch of memristor crossbar arrays^26^. A simple estimation shows that firing one of the devices can locally rise temperature up to 5 K in the other one, enough to make it fire too. By optimizing device dimensions and materials this problem could be largely avoided, and such temperature increment could be limited to a few mK (see methods). Another potential drawback is that, due to the proximity to a phase transition, temperature must be precisely controlled when working with IMT-based neuristors. Although this could be hard to implement in very large circuits, it could also be a positive feature. The human brain operates close to criticality and it is only functional in a very narrow temperature range^47^. It has been argued that this critical behaviour is what allows to perform the complex cognitive task of an intelligent system^48^. In this sense, working at the edge of a phase transition might be an ideal platform to explore new and more complex phenomena in neuromorphic computing.

Caloritronics and resistive switching can be combined to create scalable and autonomous neuristors. We demonstrated four basic neural functionalities: activity, volatility, leaky integrate-and-fire dynamics and rate coding using simple devices that can be downscaled well below the μm scale. Combined with the fast advances in memristor technology, this could pave the way to develop dense neuromorphic hardware, allowing for deeper and more complex neural networks. Our approach could be generalized to other physical phenomena. Other systems at the edge of a phase transition are very sensitive to external stimuli and might show a similar behaviour. On a broader scope, we show that, although often regarded as an undesirable consequence, power dissipation might actually enable new ways of computing, by taking advantage of the rich phenomenology of correlated systems.

## Methods

### Sample preparation

A 70 nm VO_2_ film was grown by reactive sputtering on top of an R-cut Al_2_O_3_ substrate. A 4 mtorr Argon/Oxygen mix (8% O_2_) was used during deposition. The substrate temperature was kept at 520 °C, and cooled down after sputtering at a rate of 12 °C/min. X-ray diffraction shows textured orientation along 〈100〉 for VO_2_. Transport measurements show a four orders of magnitude IMT, confirming the high quality of the film. The device was fabricated in two lithographic steps (layers). In the first layer, e-beam lithography end e-beam evaporation was used to pattern two Ti (20 nm)/Au (30 nm) electrodes. A small gap (~50 nm) was left between both electrodes, so large electric fields could be generated by applying a few volts. The second layer consists on an Al_2_O_3_ (70 nm)/Ti (20 nm)/Au (30 nm) nanowire, patterned on top of the gap and running perpendicular to the first layer electrodes. E-beam lithography and e-beam evaporation was used for this purpose. Several of such devices are patterned in a single sapphire substrate. Optical lithography and reactive ion etching was used to remove the VO_2_ outside of the gap area and isolate the different devices from each other. More information on the device fabrication process can be found in Supplementary Figure S2.

### Fast transport measurements

Measurements were carried out in a TTPX Lakeshore cryogenic probe station. The station is equipped with high-speed (20 GHz) probes, with ground/line/ground geometry and 50 Ω characteristic impedance. In order to avoid reflections (the insulating resistance of the device is in the 10^4^ Ω range) a 50 Ω termination to ground was installed before the sample. A 240 MHz Tektronix function generator was used to create the voltage pulses and a 50 Ω terminated Tektronix broadband oscilloscope (20 GHZ) was used to monitor the current. The electrical circuit set up ensured a rise time around 5 ns.

### Simulations of the device dynamics

A simple, lumped-element model was used to investigate the electro-thermal dynamics of the device (Fig. 5a,b). The electrical part of the model considers a VO_2_ gap as a resistor in parallel with a capacitor *C*, playing the role of parasitic capacitance of the circuit. The charge accumulated in such capacitor is *Q*. A load resistor *R*_*L*_ is placed in series, and *V*_*DC*_ constant bias voltage is applied across the gap and the load. We label the total current as *I*, while *I*_*R*_ and *I*_*C*_ are the currents flowing through the VO_2_ and the capacitor. The heating resistor *R*_*Heater*_ is electrically isolated from the rest of the circuit, with an input current *I*_*Input*_ passing through it.

The value *R* depends on the VO_2_ state: metallic or insulating. *R* = *R*_*met*_ = 200 Ω in the metallic state, while *R*_*ins*_ = *α*⋅e^*β/T*^ in the insulating state. The values of *α* = 0.0178 and *β* = 4500 are such that *R*_*ins*_ = 10 kΩ at 339 K, and *R*_*ins*_ = 58.4 kΩ at 300 K. Whether VO_2_ is metallic or insulating depends on the device current temperature as well as its thermal history. A hysteresis is set between 335 K and 339 K to mimic the first-order nature of the IMT.

At each simulation step *i*, *R*[*i*] is evaluated depending on the thermal history, and the current through the gap and the capacitor are calculated:

$${I}_{gap}[i]=Q[i]/(R[i]\cdot C)$$

$${I}_{C}[i]=({V}_{DC}-{I}_{gap}[i]\cdot R[i])/{R}_{L}-{I}_{gap}[i]$$

With this, we can calculate the evolution of Q using the Euler method.

$$Q[i+1]=Q[i]+{I}_{C}[i]\cdot \delta t$$

where δ*t* is the simulation step.

The temperature T is governed by the thermal part of the model. The model considers the VO_2_, the Al_2_O_3_ barrier and the heating element as a single system with homogeneous temperature. Although simple, it accurately mimics the experimental results. The are two heat sources: Joule heating in the gap (${Q}_{gap}=R\cdot {I}_{gap}^{2}$) and in the heater (${Q}_{Heater}={R}_{Heater}\cdot {I}_{Input}^{2}$). Heat is evacuated from the device into the environment, consisting on the metallic pads and the sapphire substrate. Such heat loss will depend on the temperature difference between the device and the base temperature of the environment *T*_*Base*_.

The temperature evolution is calculated using the Euler method:

$$T[i+1]=T[i]+\frac{1}{{C}_{th}}\cdot (R\cdot {I}_{gap}^{2}+{R}_{Heater}\cdot {I}_{Input}^{2}-{S}_{th}\cdot (T[i]-{T}_{Base}))\cdot \delta t$$

where *C*_*th*_ and *S*_*th*_ are the thermal capacitance and conductance of the system respectively.

We must note that for simplicity we consider the powers dissipated in the gap and the heater to contribute equally to the temperature change in the VO_2_. We treat the whole neuristor as a single thermal element with the same temperature.

By considering the individual thermal capacitances of the VO_2_, Ti/Au pads and Al_2_O_3_ barrier enclosed in the 400 nm × 400 nm area of the device, we estimated ${C}_{th}\approx 1.3\cdot {10}^{-13}J/K$. For this calculation we used the following density *d* and specific heat *c* values^49,50^: $\,{d}_{V{O}_{2}}=4.34\cdot {10}^{3}\frac{kg}{{m}^{3}}$, ${c}_{V{O}_{2}}=690\frac{J}{K\cdot kg}$, ${d}_{Au}=19.3\cdot {10}^{3}\frac{kg}{{m}^{3}}$, ${c}_{Au}=129\frac{J}{K\cdot kg}$, ${d}_{Ti}=4.54\cdot {10}^{3}\frac{kg}{{m}^{3}}$, ${c}_{Ti}=523\frac{J}{K\cdot kg}$, ${d}_{A{l}_{2}{O}_{3}}=3.97\cdot {10}^{3}\frac{kg}{{m}^{3}}$ and ${c}_{A{l}_{2}{O}_{3}}=854\frac{J}{K\cdot kg}$

To estimate the thermal conductance, we took into account two contributions: the thermal conductance of the metallic pads, and the vertical thermal conductance through the substrate. The most thermally resistive part of the pads is the 1.5 μm long stretch closer to the device center. Considering only this part of the pads, we estimated ${S}_{pads}\approx 1.3\cdot {10}^{-5}\,W/K$. Vertical heat transport will go through the VO_2_ into the sapphire substrate and we estimate it to be ${S}_{vertical}\approx 8.5\cdot {10}^{-6}W/K$. This gives a total conductance value ${S}_{th}\approx 2.2\cdot {10}^{-5}W/K$.

For this calculation we used the following thermal conductivity values^49,50^: ${\sigma }_{V{O}_{2}}=6\frac{W}{m\cdot K}$, ${\sigma }_{Au}=310\frac{W}{m\cdot K}$, ${\sigma }_{Ti}=21.9\frac{W}{m\cdot K}$ and ${\sigma }_{A{l}_{2}{O}_{3}}=30\frac{W}{m\cdot K}$.

The effect of downsizing and material choice can be explored by calculating the thermal conductance of a similar device in which Au is substituted by Ti as metallic contact, a TiO_2_ substrate is used, the pad size has been reduced one order of magnitude (down to 40 nm), while the thickness has been decreased to half. Using^50^ ${\sigma }_{Ti{O}_{2}}=9\frac{W}{m\cdot K}$, we get:

$${S}_{vertical}\approx 9.4\cdot {10}^{-8}W/K,{S}_{pads}\approx 3.7\cdot {10}^{-7}W/K,{S}_{Total}\approx 4.6\cdot {10}^{-7}W/K$$

This is a two orders of magnitude reduction in heat leakage towards the environment, which allows reducing the Joule heating.

Such changes would also decrease the specific heat of the system to ${C}_{th}\approx 1.3\cdot {10}^{-13}J/K$. This would increase the ratio ${S}_{th}/{C}_{th}$, and hence, the system dynamics by a factor of 4.5.

#### Parameters used in the simulation

Despite the simplicity of the model, we observe spiking patterns very similar to the experiments. Although the parameters were adjusted to observe oscillatory behavior, they were kept as close as possible to the device characteristics:

*T*_*Base*_ = 325 K, *V*_*DC*_ = 4.4 (Fig. 5d)–5.5 V (Fig. 5c), *R*_*ins*_ (339 K) = 10 kΩ, *R*_*met*_ = 200 Ω, *R*_*L*_ = 2.0 (Fig. 5d) – 5.0 kΩ (Fig. 5c), *I*_*Input*_ = 0–5 mA (variable), *C* = 0–10^−10^ F (variable), *R*_*Heater*_ = 20 Ω, *C*_*th*_ = 10.10^−13^ J/K, *S*_*th*_ = 10.10^−5^ W/K, *T*[0] = 325 K, *Q*[0] = 0 C and ∂*t* = 10^−13^ s.

### Estimation of temperature rise outside of the neuristor

An estimation of the temperature rise can be obtained by considering the heat flow into a substrate coming from a point source at the surface. In this case, the point source is the proposed neuristor. In an isotropic case, the temperature at a distance *r* will be given by:

$$T={T}_{Base}+\,Q/2\pi r{\sigma }_{substrate}$$

where $Q={S}_{Th}\cdot ({T}_{Neuristor}-{T}_{Base})$ is the total heat flow coming from the device.

In our experimental device, we estimated ${S}_{Th,vertical}\approx 8.5\cdot {10}^{-6}\,W/K$. According to our simulations, for a parasitic capacitance *C* = 10^−10^ F, *T*_*Neuristor*_ rises 100 K during a spike. Such spike would increase the temperature 5 K at in a point 1 μm away from the device. With the optimization proposed in the simulations part of this methods section, ${S}_{Th,vertical}\approx 9.4\cdot {10}^{-8}\,W/K$. For a system with no parasitic capacitance, the simulations show that *T*_*Neuristor*_ rises just 10 K during a spike. Such spike would increase temperature just 15 mK in a point 1 μm away.

We must point out that this calculation is approximate and does not take into account factors such as cooling from the contact pads, which will depend on the particular device design. We must also note that this estimation is an upper limit to the temperature increment, since it considers a steady state in which the device is constantly at the maximum temperature that it reaches during firing. We expect the actual temperature change to be lower.

## Supplementary information

Supplementary Information.

## Data Availability

The data supporting the plots and claims of this manuscript are available from the corresponding authors upon reasonable request.
