# Supplementary material for: A caloritronics-based Mott neuristor
Source: Sci Rep. 2020 Mar 9;10:4292. doi: 10.1038/s41598-020-61176-y (PMC7062821; doi:10.1038/s41598-020-61176-y)
Supplement: Supplementary file 1 — Supplementary Information. [file 41598_2020_61176_MOESM1_ESM.pdf]

Supplementary Information

A caloritronics-based Mott neuristor

Javier del Valle*, Pavel Salev, Yoav Kalcheim and Ivan K. Schuller

Department of Physics and Center for Advanced Nanoscience, University of California-San Diego, La Jolla, California 92093, USA

*Corresponding author: jdelvallegranda@physics.ucsd.edu

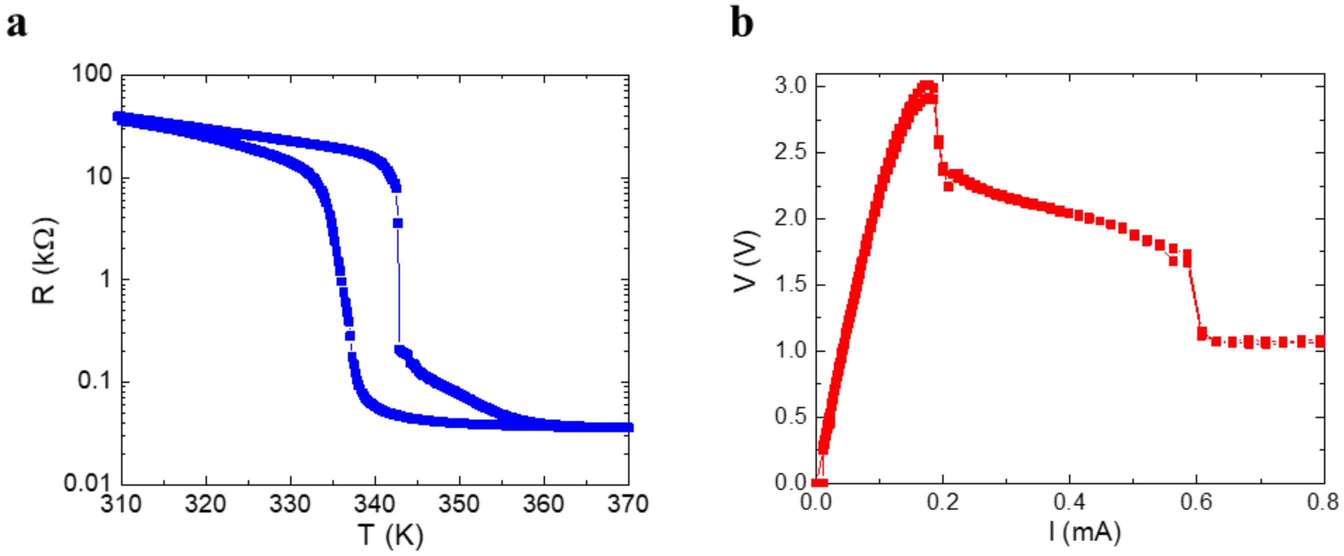

Supplementary Figure S1 | Static properties of a VO_2 nanogap. a R vs T. A hysteretic, first order insulator to metal transition is observed. Discontinuous jumps in the resistance indicate switching of individual metallic/insulating domains. **b** Current-controlled I-V characteristic of VO_2 gap at T=320 K. A sudden decrease of resistance can be observed at the current above 0.19 mA indicating electrically-driven IMT.

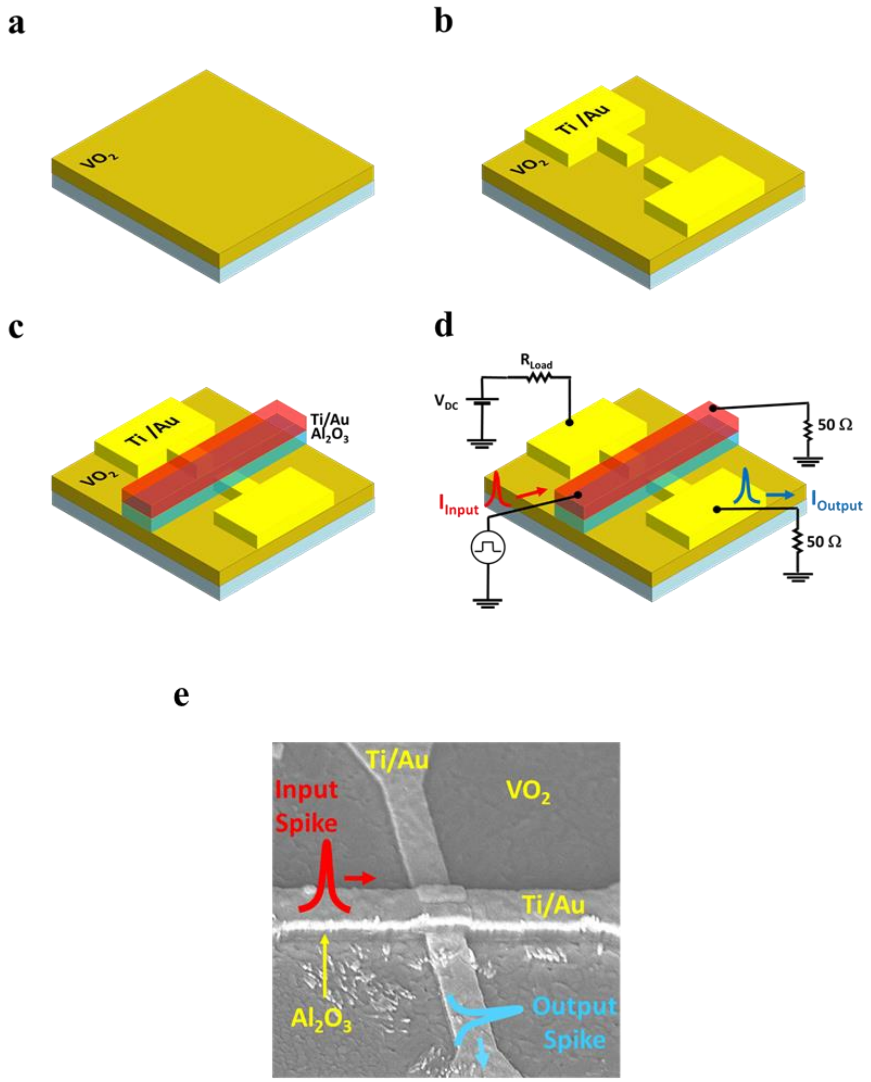

Supplementary Figure S2 │ Fabrication and configuration of the neuristor. a-d different stages of the fabrication and set up process. **a** shows the VO_2 film (light brown) on top of a sapphire substrate (light blue). **b** shows the sample after a first lithographic step in which two Ti/Au electrodes (yellow) are patterned, leaving a small gap in between (~50 nm). **c** shows the sample after the second lithographic step in which the following trilayer was grown: Al_2O_3 (70 nm, blue) / Ti (20 nm, red) / Au (30 nm, red). **d** shows the electrical connections used for the fast transport measurements. **e** Tilted SEM image of the device. The Al_2O_3 and metallic layers of the heating electrode are visible.
